# Learning Words While Listening to Syllables: Electrophysiological Correlates of Statistical Learning in Children and Adults

**DOI:** 10.3389/fnhum.2022.805723

**Published:** 2022-02-23

**Authors:** Ana Paula Soares, Francisco-Javier Gutiérrez-Domínguez, Alexandrina Lages, Helena M. Oliveira, Margarida Vasconcelos, Luis Jiménez

**Affiliations:** ^1^Human Cognition Lab, CIPsi, School of Psychology, University of Minho, Braga, Portugal; ^2^Psychological Neuroscience Lab, CIPsi, School of Psychology, University of Minho, Braga, Portugal; ^3^Department of Psychology, University of Santiago de Compostela, Santiago de Compostela, Spain

**Keywords:** statistical learning, speech segmentation, transitional probability, developmental changes, implicit learning, explicit learning, implicit statistical learning, electrophysiological correlates

## Abstract

From an early age, exposure to a spoken language has allowed us to implicitly capture the structure underlying the succession of speech sounds in that language and to segment it into meaningful units (words). Statistical learning (SL), the ability to pick up patterns in the sensory environment without intention or reinforcement, is thus assumed to play a central role in the acquisition of the rule-governed aspects of language, including the discovery of word boundaries in the continuous acoustic stream. Although extensive evidence has been gathered from artificial languages experiments showing that children and adults are able to track the regularities embedded in the auditory input, as the probability of one syllable to follow another syllable in the speech stream, the developmental trajectory of this ability remains controversial. In this work, we have collected Event-Related Potentials (ERPs) while 5-year-old children and young adults (university students) were exposed to a speech stream made of the repetition of eight three-syllable nonsense words presenting different levels of predictability (high vs. low) to mimic closely what occurs in natural languages and to get new insights into the changes that the mechanisms underlying auditory statistical learning (aSL) might undergo through the development. The participants performed the aSL task first under implicit and, subsequently, under explicit conditions to further analyze if children take advantage of previous knowledge of the to-be-learned regularities to enhance SL, as observed with the adult participants. These findings would also contribute to extend our knowledge of the mechanisms available to assist SL at each developmental stage. Although behavioral signs of learning, even under explicit conditions, were only observed for the adult participants, ERP data showed evidence of online segmentation in the brain in both groups, as indexed by modulations in the N100 and N400 components. A detailed analysis of the neural data suggests, however, that adults and children rely on different mechanisms to assist the extraction of word-like units from the continuous speech stream, hence supporting the view that SL with auditory linguistic materials changes through development.

## Introduction

Although a large number of studies have shown that the ability to extract regularities from the sensory environment, an ability known as statistical learning (SL; Saffran et al., 1996), is observed in young children (e.g., Saffran et al., 1996; Teinonen et al., 2009; Arciuli and Simpson, 2011; Bertels et al., 2015; Bosseler et al., 2016; Choi et al., 2020) and adults (e.g., Saffran et al., 1997; Fiser and Aslin, 2002; Turk-Browne et al., 2009; Johnson et al., 2020), little is known about how this ability changes through development.

This occurs, at least in part, because early works on SL, as well as in the implicit learning (IL)-related field (see Christiansen (2019)), claimed that SL/IL is an early-maturing ability that remains quite stable across development as no differences in performance had been observed between children and adults in those pioneering works (e.g., Reber, 1989, 2013; Saffran et al., 1997). However, a growing body of research conducted in the last decade has challenged this view by showing not only that SL/IL improves with age (see Lukács and Kemény (2015), Zwart et al. (2017), Arnon (2020) for reviews) but also that the developmental trajectory of this ability might not be the same across sensory modalities and types of stimuli (e.g., Raviv and Arnon, 2018; Shufaniya and Arnon, 2018). For instance, Raviv and Arnon (2018), using auditory syllables and visual figures in auditory (aSL) and visual (vSL) SL tasks modeled from Saffran et al. (1996), showed that while vSL improved in children aged 5–12 years old, aSL did not. This could account for the disparity of results found in the previous studies showing age differences for vSL (e.g., Arciuli and Simpson, 2011; Bertels et al., 2015), but not for aSL (e.g., Saffran et al., 1997; see however Emberson et al. (2019) for SL improvements in both modalities). Nonetheless, in a subsequent work, Shufaniya and Arnon (2018) showed that the absence of age differences in the aSL was not due to the sensory modality *per se* but rather to the type of stimuli used. Indeed, when instead of auditory syllables the authors used familiar sounds in the aSL task, Shufaniya and Arnon (2018) found evidence of SL improvements in children in both modalities. These findings strongly suggest that SL is not age-invariant, as claimed by earlier works (Reber, 1989, 2013; Saffran et al., 1997), except for auditory linguistic materials. They also agree with other works claiming, on one hand, that the extraction of regularities from the speech environment is a powerful mechanism for language acquisition (see Romberg and Saffran, 2010), and, on the other hand, that against what occurs in most cognitive skills, adults are not better than children at learning new languages (Thiessen et al., 2016; Smalle et al., 2017).

Nevertheless, it should be noted that most of these findings were obtained through laboratory experiments, in which the learning of the regularities was only assessed after the exposure phase by using behavioral recognition tasks. Indeed, following the paradigm introduced by Saffran et al. (1996) in the late 1990s, in those studies, participants were first exposed to a continuous stream made of repetitions of stimuli (e.g., syllables, familiar sounds, and figures) which, unbeknownst to them, were grouped into triplets (e.g., “*tokibu*,” “*tipolu*,” “*gopila*”). The triplets always appear together in the stream with no pauses between each other (e.g., “*tokibutipolugopilatokibu*”) and without any information about the task or the stimuli (i.e., under incidental or implicit conditions). After exposure, the participants were presented with pairs of triplets (a nonsense word presented during the familiarization phase vs. a foil made of the same syllables but presented in a new sequence – “*tokibu*” vs. “*tokopi*”) and asked to identify which one most resembles the stream presented before, i.e., to perform a two-alternative forced-choice (2-AFC) task. If performance exceeded the chance level, SL was assumed to have occurred as only the tracking of the statistical regularities – typically the likelihood of two stimuli following one another [transitional probability (TP)] – allowed correct discrimination. Note that, in this paradigm, TPs within triplets are typically higher (usually of 1) than TPs across triplets’ boundaries (usually of 0.33). This means that, within a triplet, a given syllable is always preceded by another given syllable, whereas, across triplets, a given syllable can be followed by any other syllable that begins the remaining triplets (see also Lukács and Kemény (2015) for evidence on developmental changes of IL using other behavioral paradigms).

Despite the widespread use of the 2-AFC task in research, its suitability to assess SL has been increasingly questioned (Erickson et al., 2016; Siegelman et al., 2017, 2018b; Frost et al., 2019), particularly when used with young participants (Bertels et al., 2015; van Witteloostuijn et al., 2019; Arnon, 2020; Lukács et al., 2021). Besides involving the use of a small number of 2-AFC trials, raising important psychometric concerns (see Siegelman et al. (2017), Soares et al. (2021c)), it is also worth noting that the 2-AFC task relies on explicit judgments, which are largely dependent on other high-order cognitive skills (e.g., decision-making processes) that could not be fully developed in young children (see Lukics and Lukács (2021) for similar arguments). In addition, the 2-AFC task is an offline post-learning task that only measures the *result* of the learning that presumably has taken place in the previous familiarization phase, and not the *processes* underlying that result (Batterink and Paller, 2017; Siegelman et al., 2017; Soares et al., 2020). Hence, it is possible that, even though children and adults might not differ in terms of aSL outcomes, they might, nevertheless, differ in the mechanisms they recruit to assist SL at each developmental stage. Further research using other tasks and techniques is thus required to get a deeper understanding of how aSL might change across development. In particular, the use of Event-Related Potentials (ERPs) is highly recommended as it allows measuring learning as the exposition to the speech stream unfolds with millisecond precision even in the absence of any behavioral response (see Daltrozzo and Conway (2014)). Although these factors make ERPs an exceptional tool to make meaningful comparisons of SL across the life span, studies examining this issue using this technique are scarce. As far as we know, only Jost et al. (2015) used a variation of the oddball paradigm, called a novel predictor-target paradigm, to explore the development of the neural mechanisms that support the extraction of regularities embedded in a continuous stream made of a succession of visual stimuli with adults and children aged 6–9 and 9–12 years old. In this task, the participants were asked to press a button whenever a given target (a colored circle) appeared at the center of the computer screen. Unbeknownst to them, the target was predicted by colored circles with varying degrees of probability (high, low, and null). Results showed an enhanced P300 in the three groups of the participants for the high relative to the low or null predictors, hence providing evidence for the invariance model of SL at the neural level.

Other ERP studies conducted either with adults (Sanders et al., 2002, 2009; Cunillera et al., 2006, 2009; De Diego Balaguer et al., 2007; Abla et al., 2008; François et al., 2014; Mandikal-Vasuki et al., 2017a; Soares et al., 2020) or children (Teinonen et al., 2009; Bosseler et al., 2016; Mandikal-Vasuki et al., 2017b; Choi et al., 2020; Pierce et al., 2021) have provided, however, evidence for developmental changes in the electrophysiological correlates of SL. Specifically, the EEG data collected with the adult participants during the exposure phase of a triplet embedded task modeled from Saffran et al. (1996) suggest the N100 and, particularly, the N400 ERP components as the neural signatures of online segmentation in the brain. The auditory N100 has been associated with the processing of the sensory features of stimuli and predictive mechanisms involved in the processing of speech streams (e.g., Heinks-Maldonado et al., 2005). Additionally, the N400 has been proposed to reflect processes related to the processing of the stream into perceptual units beyond the syllable unit *per se* (i.e., building up of “word” prototypes). Note that, although the N400 has been classically associated with the difficulty of retrieving information from semantic memory (Holcomb et al., 1992; Kutas and Federmeier, 2011; Brouwer et al., 2012), in artificial language paradigms that rely on the use of pseudowords, which are, by definition, meaningless, larger N400 amplitudes have been observed not only for less predictable than for more predictable positions of a triplet (initial vs. final) but also for triplets presenting high than low levels of predictability (e.g., Abla et al., 2008; François et al., 2014; Mandikal-Vasuki et al., 2017a; Soares et al., 2020). These findings suggest modulations in the N400 component to be associated in artificial learning paradigms both with predictive mechanisms and facilitated access and/or more successful integration of triplets into perceptual units in long-term memory(see Lau et al. (2008) for a review of how modulations in the N400 respond to different paradigms), a finding consistent with the interpretation of the N400 as an index of the emergence of a pre-lexical trace of “words” in the brain (e.g., Sanders et al., 2002; Cunillera et al., 2006, 2009; De Diego Balaguer et al., 2007; Batterink and Paller, 2017; Soares et al., 2020).

Evidence for online segmentation in the brain has also been found with children participants (Teinonen et al., 2009; Bosseler et al., 2016; Mandikal-Vasuki et al., 2017b; Choi et al., 2020; Pierce et al., 2021). For instance, Mandikal-Vasuki et al. (2017b), exploring whether children aged 9–11, with and without musical training, differ in the neural correlates of SL when exposed to auditory (tones) and also visual streams (cartoon figures), showed both groups to present larger amplitudes in the P100 and N250 components for the less vs. more predictable positions in the case of the auditory triplets, and the P100, N200, and P300 components in the case of visual triplets. Differences across groups were only observed in the auditory domain in the N250 components reflected in a larger amplitude for the musician than for the non-musician group, a result also observed by Mandikal-Vasuki et al. (2017a) in a study conducted with adult participants but in different (N100 and N400) time windows. Additionally, Pierce et al. (2021), in a recent study analyzing whether maternal stress was associated with the neural responses of aSL using tones as stimuli in 26-month-old children, found evidence of online segmentation in the P200 component.

Although all these studies seem to point toward the existence of developmental changes in the electrophysiological correlates of SL, the fact that they have relied on different stimuli (e.g., tones and syllables) and populations (either adults or children) makes it difficult to draw any conclusions about the developmental changes of the processes underlying SL. The use of the same task with the same stimuli, as in Jost et al.’s (2015) work, is highly recommended as only it allows to make direct comparisons across groups and to further ascertain whether the neural processes underlying aSL with linguistic stimuli are developmentally invariant or not. It is also important to emphasize that, although the vast majority of the aSL studies with linguistic materials have used three-syllable nonsense words presenting the same level of predictability (i.e., TP of 1.00 – see, however, Bogaerts et al. (2016), Siegelman et al. (2018a), Tsogli et al. (2019), Johnson et al. (2020), Soares et al. (2020, 2021a,2021b,2021c), Gutiérrez-Domínguez et al. (2021), Lages et al. (2021)), studying how SL works under less predictable conditions is equally important. As Soares et al. (2020) have recently pointed out, in natural languages, syllables, as well as other linguistic units (e.g., phonemes, morphemes, and words), do not follow each other with 100% of certainty (syllables as/*cur*/occur in different words and syllable positions, such as in/*cur*.va.ture/, /in.*cur*.sion/, or /re.oc.*cur*/). Using nonsense words with different TPs can be thus highly beneficial. It can contribute to increase the variance along which SL can be measured, to mimick what occurs in natural languages closely, and, importantly, to increase the chances of age-related differences in the processes recruited to assist SL to be observed.

Finally, although most studies have tested SL under incidental conditions, which have been used to support the view that SL works in an automatic and non-conscious manner, recent studies have shown that both implicit and explicit learning mechanisms might be involved in SL (Batterink L. et al., 2015; Batterink L.J. et al., 2015; Bertels et al., 2015). Batterink L.J. et al., 2015 found evidence for explicit knowledge during aSL, even when no explicit instructions were provided to the participants to perform the task (see also Jiménez et al. (2020) and Soares et al. (2021d) for recent evidence with the artificial grammar learning paradigm). Further support for the involvement of explicit learning mechanisms in SL comes from neuroimaging studies, showing that responses to statistical regularities are observed in areas generally associated with implicit (e.g., basal ganglia) and explicit (e.g., medial-temporal areas, including hippocampus) structures (Turk-Browne et al., 2009; Karuza et al., 2013), in accordance with the two-memory learning systems (procedural vs. declarative) model in the brain (see Batterink et al. (2019) for a review). However, it is possible that the recruitment of these systems to assist SL might change across the life span as procedural learning (implicit) seems to rely mainly on brain networks that mature early in life, whereas declarative learning (explicit) recruits cortical structures that improve with age. Studies examining how the recruitment of these structures might change across development and affect SL are yet scarce. Previous studies dissociating these two types of knowledge through the manipulation of the instructions (implicit vs. explicit) with adult participants have shown that the previous knowledge of the to-be-learned regularities in a triplet-embedded task enhanced 2-AFC performance, particularly when the instructions provided were specific enough (Batterink L.J. et al., 2015; Soares et al., 2020). At the neural level, explicit instructions have also been shown to produce a reduction in the P300 to target syllables in a post-learning detection task (Batterink L.J. et al., 2015) and a reduction in the N250 component to “words” presented under explicit vs. implicit conditions (Soares et al., 2020). These findings were interpreted as facilitation due to the involvement of controlled and effortful processes in SL. Studies examining if SL can be enhanced through the use of explicit instructions in children remain to be conducted.

## Current Study

This work aimed to get new insights into the age-related differences of aSL with linguistic stimuli (syllables) by relying, on one hand, on an online technique (ERPs) directed to overcome the limitations of previous studies based on behavioral SL outcomes (particularly the 2-AFC task), and, on the other hand, on more complex speech streams combining three-syllable nonsense words with different levels of predictability (high vs. low). The speech stream was presented to 5-year-old children and young adults (university students) under implicit and explicit learning conditions to further analyze whether children can take advantage of the previous knowledge of the to-be-learned regularities to enhance SL, as observed in previous studies with adult participants. Against previous works (Batterink L. et al., 2015; Batterink L.J. et al., 2015), the manipulation of instructions (implicit vs. explicit) was done in a within-subject design to minimize the role of individual differences in the results (see Siegelman et al. (2017)). Moreover, the temporal changes of the neural responses to the speech streams during familiarization were also analyzed to further investigate whether children and adults showed neurofunctional differences in the amount of exposure they need to unravel the statistical structure embedded in the input (see Abla et al. (2008), François et al. (2014), Batterink and Paller (2017), Soares et al. (2020) for examples).

Based on the reviewed literature, we expected that if the processes underlying aSL with linguistic materials were early maturing and stable across development, as previous behavioral works suggest (e.g., Saffran et al., 1997; Raviv and Arnon, 2018; Shufaniya and Arnon, 2018), no differences should be observed in the electrophysiological correlates indexing SL in the brain, like the N100 and the N400 ERP components, even though slight differences might be observed due to differences in topography, amplitude, and latency arising from development and maturation factors (Albrecht et al., 2000; Pang and Taylor, 2000; Junge et al., 2021). In contrast, if aSL with linguistic materials elicited different neural responses in children and adults, as previous studies conducted either with adults or children suggest (e.g., Cunillera et al., 2006, 2009; Abla et al., 2008; Bosseler et al., 2016; Mandikal-Vasuki et al., 2017a,b; Choi et al., 2020; Soares et al., 2020; Pierce et al., 2021), distinct modulations in the N100 and the N400 components should be observed. This later result would provide further evidence for neurodevelopmental changes in the processes recruited to assist aSL with linguistic materials, even though differences at a behavioral (2-AFC) level might not be noticed. It would be also possible that, even if the same basic pattern of neural results emerged, differences in the temporal dynamics of SL might be observed, with earlier effects for adults than children. In particular, we expected to replicate previous findings with adult participants (Abla et al., 2008; François et al., 2014; Mandikal-Vasuki et al., 2017a; Soares et al., 2020), namely high-predictable “words” eliciting larger N400 amplitude than low-predictable “words,” and “words” presented under explicit conditions eliciting a reduced N100 amplitude than “words” presented under implicit conditions, indexing attentional (top-down) effects. As exposure to the speech streams unfolded, we also expected an enhancement in the N100 component, indexing the involvement of predictive mechanisms, and in the N400 reflecting the formation of a pre-lexical trace of “words” in the brain.

## Materials and Methods

### Participants

Twenty-four children (13 female, *M*_age_ = 5;7; range, 5;1 to 6;5) from Portuguese kindergarten institutions and 24 students (22 female, *M*_age_ = 20;3; range, 18;1 to 31;2) from the University of Minho participated in the study. All the participants were native speakers of European Portuguese, with normal hearing and no reported history of learning or language disabilities and/or neurological problems. All were right-handed, as assessed by the Portuguese adaptation of the Edinburgh Handedness Inventory (Oldfield, 1971; Espirito-Santo et al., 2017). Written informed consent was obtained from each adult participant and parents/legal representatives in the case of children participants. The study was carried out in accordance with the guidelines of the Declaration of Helsinki and approved by the ethics committee of the local Ethics Committee (University of Minho, SECSH 028/2018). Seven participants (four children and three adults) were excluded from the EEG and also from the behavioral analyses due to artifact rejection.

### Stimuli

Sixteen three-syllable nonsense words taken from Soares et al.’s (2020) were used in the implicit and explicit versions of the aSL tasks (eight “words” per task). The nonsense “words” were made of 32 unique European Portuguese syllables evenly distributed across two syllabaries (Syllabary A and Syllabary B). Words from each syllabary were used either in the implicit or explicit aSL tasks (counterbalanced across the participants) to avoid carry-over effects. Each syllable had duration of 300 ms. Syllables were concatenated into triplets with a 50-ms interval between each other (1,050 ms per “word”) using the Audacity^®^ software (1999–2019). In each syllabary, four “words” presented TPs between syllables within a “word” of 1.00 (high-TP “words”), whereas the remaining four presented TPs within a “word” of 0.33 (low-TP “words”). For instance, the nonsense word “*tucida*” from Syllabary A and the nonsense word “*todidu*” from Syllabary B correspond to high-TP “words” as the syllables they entail only appear in those “words” and in those specific syllable positions, while the nonsense word “*dotige*” from Syllabary A and the nonsense word “*pitegu*” from Syllabary B correspond to low-TP “words” as the syllables they entail appear in three different “words” at different (initial, medial, and final) syllable positions (“*tidomi*,” “*migedo*,” and “*tepime*,” “*megupi*,” respectively – see Table 1 for other examples).

**TABLE 1:** Three-syllable nonsense words and foils from Syllabary A and Syllabary B.

Transitional probabilities	Syllabary	Stimuli	
		“Words”	Foils
High-TP	A	*tucida*	*tumica*
		*bupepo*	*bugego*
		*modego*	*modopo*
		*bibaca*	*bitida*
	B	*todidu*	*tomeco*
		*cegita*	*cegube*
		*gapabe*	*gapita*
		*bomaco*	*botedu*
Low-TP	A	*dotige*	*dobage*
		*tidomi*	*tidemi*
		*migedo*	*mipedo*
		*gemiti*	*geciti*
	B	*pitegu*	*pimagu*
		*tepime*	*tepame*
		*megupi*	*megipi*
		*gumete*	*gudite*

**FIGURE 1:**
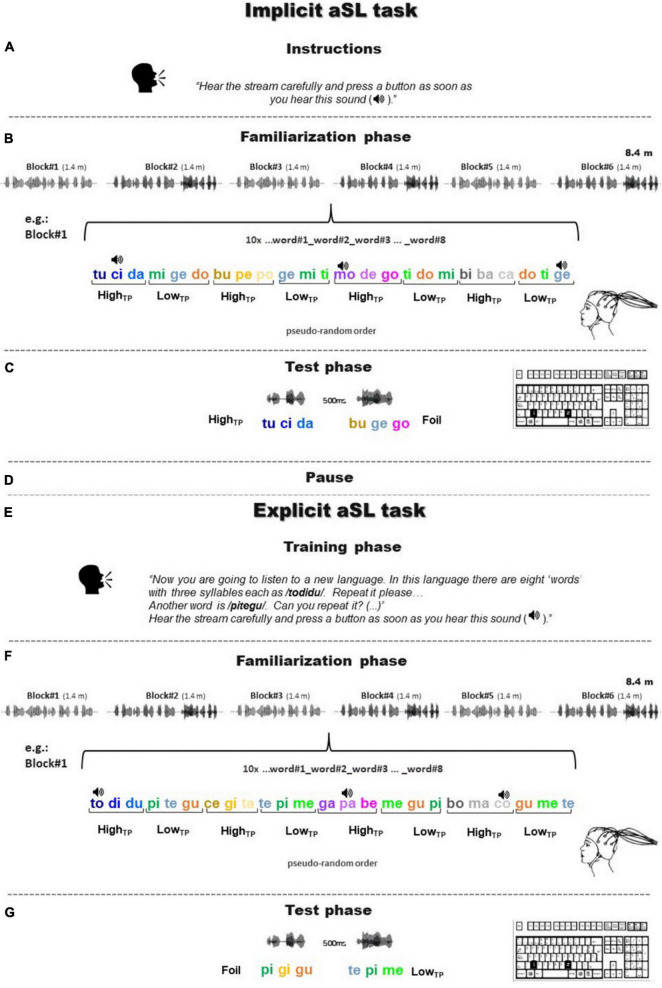
A visual summary of the experimental design. Panels **(A–G)** illustrate the timeline of the experimental procedure in which the implicit and, subsequently, the explicit aSL tasks were administered. Each aSL task comprised three parts: instructions, familiarization phase, and test phase. Each task was initiated with specific instructions **(A,E)** that determined the conditions under which the aSL task was performed: **(A)** implicit instructions (i.e., without knowledge of the stimuli or the structure of the stream) or **(E)** explicit instructions (i.e., with explicit knowledge or pre-training on the “words” presented in the stream). In the familiarization phase of both tasks **(B,F)** during which EEG data were collected, the participants were presented with a continuous auditory stream of four high-TP and four low-TP “words,” with chirp sounds (depicted as a speaker icon in the figure) superimposed over specific syllables. The chirp sounds could emerge at any of the three syllabic positions of the “words,” which precluded its use as a cue for stream segmentation. During this phase, the participants had to perform a chirp detection task. Then, a test phase **(C,G)** consisting of a 2-AFC task asked the participants to indicate which of the two-syllable sequences (a “word” and a foil) sounded more familiar, considering the stream heard on the familiarization phase.

The nonsense words were presented in a pseudo-randomized order (the same “word” or the same syllable would never appear consecutively in a row, i.e., neither “*tidomitidomi*” situations nor “*tidomimigedo*” situations were allowed to occur). In each stream, TPs across “word” boundaries were, therefore, 0.14 for the high-TP “words,” and 0.17 for the low-TP “words.” In each stream, the “words” were presented 60 times in six blocks of 10 repetitions each (see Figure 1 ahead), lasting 8.4 min (1.4 min per block). Each speech stream was edited to include in 15% of the syllables a superimposed chirp sound (a.1-s sawtooth wave sound from 450 to 1,450 Hz) to provide the participants with a cover task (i.e., a chirp detection task) to ensure adequate attention to the stimuli during exposure. The chirp was included in all “words,” counterbalanced across syllable positions to prevent any cue for word segmentation. Correct detections in adults were 141.5 (±2.29) out of 144 in the implicit aSL task (97.8% of all responses, including false alarms) and 141.4 (±2.21) in the explicit aSL task (98.3%). Correct detections in the children group were 133.2 (±10.60) in the implicit aSL task (88.2%) and 131.2 (±5.61) in the explicit version (92.7%). In any case, differences across aSL tasks were non-significant (*p* > 0.337). These findings suggest that the participants paid appropriate and, importantly, similar attention to the speech streams presented in each of the aSL tasks, thus ruling out this factor as a potential confound.

For the 2-AFC tasks performed after the familiarization phases of the aSL tasks, we used the foils already created by Soares et al. (2020) from Syllabaries A and B (see Table 1). The foils were made up of the same syllables used in the “words,” presented with the same frequency and syllable positions as in the high- and low-TP “words.” For example, the most frequent syllables used during familiarization from Syllabary A (e.g., “*do*,” “*ti*,” “*mi*,” and “*ge”*), which appeared three times in different low-TP “words” (e.g., “*dotage*,” “*tidomi*,” “*migedo*,” and “*gemiti”*), were also presented three times in the foils (e.g., “*dobage*,” “*tidemi*,” “*mipedo*,” and “*geciti*”), whereas the less frequent syllables (e.g., “*tu*,” “*ci*,” “*da*,” “*bu*,” “*pe*,” and “*po*”), which appeared only one time in the high-TP “words” (e.g., “*tucida*,” “*bupepo*,” “*modego*,” and “*bibaca*”), were also presented one time in the foils (e.g., “*tumica*,” “*bugego*,” “*modopo*,” and “*bitida*”). However, conversely, to the syllables in the high- and low-TP “words,” the syllables in the foils were never presented together during familiarization (TPs = 0). Note, however, that due to stimuli restrictions (the number of syllables in each syllabary and the need to generate sequences of syllables never presented together before), the foils associated with the high-TP “words” entailed two syllables from the high-TP “words” and one-syllable from the low-TP “words.” The same is observed for the foils associated with the low-TP “words” that entailed two syllables from the low-TP “words” and one-syllable from the high-TP “words.” Four lists of materials were created to counterbalance syllables across positions in each syllabary. The participants in each group were randomly assigned to one list from Syllabary A and one list from Syllabary B to perform the aSL under implicit and explicit conditions with the constraint that the same number of the participants would complete a given list (six participants *per* list).

### Procedure

The participants were first presented with the implicit version of the aSL task and, subsequently, with the explicit version of an analogous aSL task (see Figure 1). In the implicit version, the participants were instructed to pay attention to the auditory stream (sequences of syllables) presented at 60 dB SPL *via* binaural headphones, because, occasionally, a deviant sound (i.e., a click) would appear, and their task would be to detect it as soon and accurately as possible by pressing the spacebar from the computer keyboard (i.e., to perform a target detection task). Following familiarization, the participants were asked to decide as accurately as possible which of two auditory stimuli (one “word” and one foil) “sounded more like” the stimuli presented before (i.e., to perform a 2-AFC task). The 2-AFC comprised 16 trials in which each of the “words” was paired with two different foils. This option was made to minimize “words” and foils repetitions as Soares et al. (2021c) have recently shown that increasing 2-AFC trials by repeating the same stimulus only increases “noise” in SL measurement. In the 2-AFC task, each trial began with the presentation of a fixation point (cross) for 1,000 ms, after which the first stimulus (“word”/foil) was presented, followed by the second stimulus. A 500-ms inter-stimulus interval separated the presentation of the stimuli. The next trial began as soon as the participants made a response or 10 s had elapsed. The 16 trials were presented in two blocks of 8 trials each. In each block, the order (first or second) by which the stimuli were presented was controlled for, so that, in half of the trials, half of the high-TP and half of the low-TP “words” were presented firstly and in the other half the reverse (counterbalanced across blocks). In each block, the high-TP and low-TP “words” were paired against half of the foils associated with each type of “word.” The trials in each block, as well as the blocks, were randomly presented to the participants.

After a brief interval, the participants underwent the explicit version of the aSL task. This version followed the same procedure adopted in the implicit aSL task, except that, previously, to the familiarization phase, the participants were presented with additional information about the stimuli that they would listen to during exposure. Specifically, the participants were told that they would be listening to some “new words” from another foreign language. Then, each of the eight new “words” was presented auditorily (one by one) to the participants and they were asked to repeat each of them correctly before the familiarization phase began. As in the implicit task, during the familiarization phase, the participants were asked to perform a target detection task (i.e., to press a button whenever they heard the click sound). After familiarization, the participants performed another 2-AFC task that mimicked the one used in the implicit version of the aSL task. The procedure took about 90 min to be completed per participant. Figure 1 depicts a visual summary of the experimental design.

### EEG Data Acquisition and Processing

Data collection was performed in an electric shielded, sound-attenuated room at Psychological Neuroscience Lab (School of Psychology, University of Minho). The participants were seated in a comfortable chair, 1 m away from a computer screen. During the familiarization phase, EEG data were recorded with a 64 channels BioSemi Active-Two system (BioSemi, Amsterdam, Netherlands) according to the international 10–20 system and digitized at a sampling rate of 512 Hz. Electrode impedances were kept below 20 kΩ. EEG was re-referenced offline to the algebraic average of mastoids. Data were filtered with a bandpass filter of 0.1–30 Hz (zero phase shift Butterworth). ERP epochs were time-locked to the nonsense words’ onset, from −300 to 1,200 ms (baseline correction from −300 to 0 ms). Independent component analyses (ICA) were performed to remove stereotyped noise (mainly ocular movements and blinks) by subtracting the corresponding components. After that, epochs containing artifacts (i.e., with amplitudes exceeding ±100 μV) were excluded. After artifact rejection, the average accepted trials by condition and group were 85% (204 trials). Only data from the participants presenting a minimum of two-thirds of trials in any condition were considered in the analyses (21 participants in the adult group and 20 participants in the children group). EEG data processing was conducted with Brain Vision Analyzer, version 2.1.1. (Brain Products, Munich, Germany).

### Data Analysis

Behavioral (2-AFC) and ERP data analyses were performed using the IBM-SPSS software (Version 27.0). For behavioral data, the% of correct responses was computed for each of the 2-AFC tasks and separately for the high-TP and low-TP “words” in each group of the participants. One-sample *t*-tests against the chance level were conducted in each group of the participants to determine whether performance in each aSL task and type of “word” was significantly different from chance (50%). ANOVA using Group (children vs. adults) as a between-subject factor and the aSL task (implicit vs. explicit) and Type of “word” (high-TP vs. low-TP) as within-subject factors were also conducted to analyze if 2-AFC performance was significantly different across groups and experimental conditions.

Individual ERPs of the familiarization phase were averaged separately per condition and aSL task. Grand averages waveforms were then calculated in each group of the participants according to the aSL task (implicit vs. explicit), Type of “word” (high-TP vs. low-TP), and length of exposure (first half: block #1, block #2, block #3 vs. second half: block #4, block #5, block #6). We chose to analyze neural data in two different parts to ensure a sufficient number of trials in each condition per participant. We have also opted to conduct the ANOVAs for the group of children and adults separately because the direct comparisons of mean amplitudes for N100 and N400 could produce effects that could arise from developmental and maturation factors and not from the manipulation of the variables, as mentioned before. Developmental changes were indexed generally by a reduction of amplitude and latency in the N100 (Albrecht et al., 2000; Pang and Taylor, 2000). Similarly, N400 was found reduced in adults, as well as other differences in latency and duration of the wave (for a recent systematic review, see Junge et al., 2021).

Based on previous aSL ERP studies (e.g., Sanders et al., 2002, 2009; Cunillera et al., 2006, 2009; De Diego Balaguer et al., 2007; Abla et al., 2008; François et al., 2014; Batterink and Paller, 2017; Mandikal-Vasuki et al., 2017b; Soares et al., 2020), mean amplitudes were measured for the following time windows, taken as the neural signatures of words’ segmentation in the brain: 80–120 ms (N100 component) for both groups; 350–450 and 400–500 ms (N400 component) for the group of adults and children, respectively. We chose a slightly later time window for the children group since data inspection revealed a longer latency of the N400 component. This delay of the N400 component in children has already been described in the literature and considered a normative evolutionary phenomenon (Juottonen et al., 1996; Hahne et al., 2004; Cummings et al., 2008). To account for the topographical distribution of the abovementioned EEG deflections, mean amplitudes’ values were obtained for the topographical regions where amplitudes were maximal: the fronto-central region of interest (ROI; F1, Fz, F2, FC1, FCz, FC2, C1, Cz, and C2) for N100 in children, and the frontal ROI (AF3, AFz, AF4, F1, Fz, F2, FC1, FCz, and FC2) and the central ROI (FC1, FCz, FC2, C1, Cz, C2, CP1, CPz, and CP2) for the rest of the cases.

Both for behavioral and ERP data, main and interaction effects that reached statistical or marginal significance levels (*p* < 0.05 or *p* < 0.08, respectively) in comparison of interest are reported. The Greenhouse–Geisser correction for non-sphericity was used when appropriate. *Post hoc* tests for multiple comparisons were adjusted with Bonferroni correction. In such cases, the *p*-values reported were the ones obtained after the Bonferroni corrections were automatically applied (i.e., the adjusted *p*-values) by the IBM-SPSS^®^ software (Version 27.0). Measures of effect size (Eta squared, $\eta_{p}^{2}$) and observed power (*pw*) for a single effect are reported in combination with the main effects of the condition.

## Results

### Behavioral Data

The mean percentages of correct responses obtained from the 2-AFC tasks performed after the exposure phases of the implicit and explicit aSL tasks per type of “word” and a group of participants are presented in Table 2.

**TABLE 2:** Mean (SD) of the number (%) of correct responses for the high- and low-TP “words” in the implicit and explicit aSL tasks per group of participants.

“Word” Group	aSL task			
	Implicit		Explicit	
	High-TP	Low-TP	High-TP	Low-TP
Children	50.1 (17.17)	45.1 (19.16)	55.7 (15.43)	52.5 (17.54)
Adults	47.1 (17.68)	58.4 (14.46)	61.4 (20.12)	65.5 (20.08)

The results from the one-sample *t*-tests against a chance level in the group of children showed that the 2-AFC performance did not differ from the chance in either of the aSL tasks and type of “words” (all *p*s > 0.115). In the adult group, the results showed that 2-AFC performance exceeded the chance level for the low-TP “words,” *t*(20) = 2.264, *p* = 0.015 in the implicit condition, and for the high-TP words, *t*(20) = 2.592, *p* = 0.017, and low-TP “words,” *t*(20) = 3.543, *p* = 0.002 in the explicit condition. These findings indicated that, in contrast to children, adults showed behavioral signs of learning in both aSL tasks and for both types of “words” except for the high-TP “words” in the implicit condition.

Moreover, the results obtained from the repeated measures ANOVA showed a main effect of group, *F*(1,39) = 4.791, *p* = 0.035, $\eta_{p}^{2}=0.109$, *pw* = 0.569, indicating, unsurprisingly, that adults outperformed children (58.1 vs. 50.8%, respectively) when both tasks were taken as a whole. A main effect of aSL task was also observed, *F*(1,39) = 11.979, *p* = 0.001, $\eta_{p}^{2}=0.235$, *pw* = 0.921. This effect indicated that the participants showed better performance in the aSL task performed under explicit than implicit conditions (58.8 vs. 50.2%, respectively) regardless of the group. Furthermore, the twofold group × type of “word” interaction was marginally significant, *F*(1,39) = 4.023, *p* = 0.052, $\eta_{p}^{2}=0.094$, *pw* = 0.499. This effect showed that, while adults tended to better recognize low-TP than high-TP “words” in both aSL tasks (62 vs. 54.3%, *p* = 0.068), in the group of children, the difference across the type of “words” failed to approach significance (48.8 vs. 52.9%, *p* = 0.338). Moreover, the interaction also revealed that adults tended to outperform children for the low-TP “words” (62 vs. 48.8%, *p* = 0.004), but not for the high-TP “words” (54.3 vs. 52.9%, *p* = 0.765).

### Event-Related Potential Data

#### N100

##### Children

In this ERP component, the ANOVA showed a main effect of the length of exposure, maximal at the fronto-central ROI, *F*(1,19) = 5.22, *p* = 0.034, $\eta_{p}^{2}=0.215$, *pw* = 0.582, indicating that, regardless of the aSL task and type of “word,” children showed a larger N100 amplitude in the second half than the first half of the aSL tasks (see Figure 4). No other main or interaction effects reached statistical significance.

**FIGURE 2:**
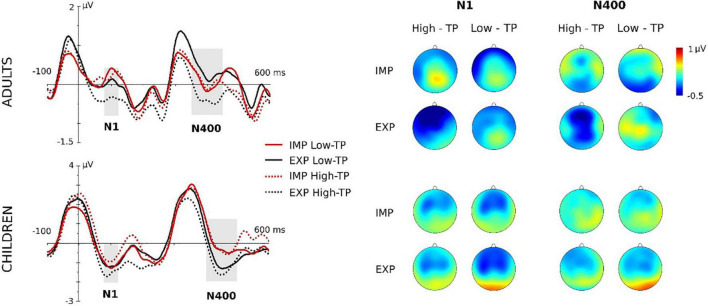
Grand-averaged waveforms (central ROI) and topographic maps for adults and children. “IMP” stands for the aSL task performed under implicit conditions, whereas “EXP” for the aSL performed under explicit instructions (first and second blocks collapsed). Gray-shaded rectangles indicate the analyzed time windows. For a better visualization of the effects, data depicted in this figure were low-pass filtered at 25 Hz after grand average.

**FIGURE 3:**
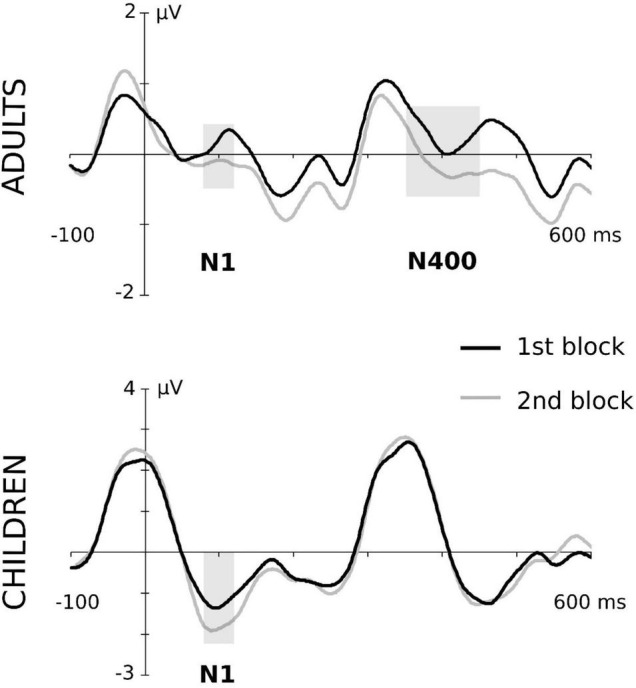
Block effects in N100 and N400 components both in the adult and children groups. Grand-averaged waveforms correspond to central ROI in adults and fronto-central ROI in children. To assure the clarity of the graphical representation, the conditions of type of “word” and aSL task were collapsed. Gray-shaded rectangles indicate the time windows in which the block effect was significant. For a better visualization of the effects, data depicted in this figure were low-pass filtered at 25 Hz after grand average.

**FIGURE 4:**
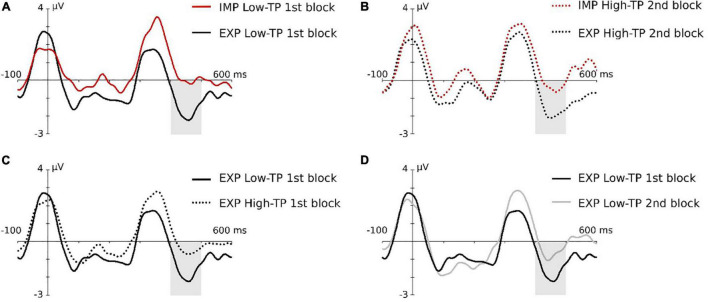
Graphical representation of the N400 triple interaction effect in the children group. Gray-shaded rectangles indicate the N400 time window. **(A)** Task effect in the low-TP condition, in the first block. **(B)** Task effect in the high-TP condition, in the second block. **(C)** Type of “word” effect under explicit instructions in the first block. **(D)** Effect of block in low-TP “words” under explicit instructions. For a better visualization of the effects, data depicted in this figure were low-pass filtered at 25 Hz after grand average.

##### Adults

Maximal effects were observed at the central ROI in this ERP component. The ANOVA showed a main effect of the aSL task, *F*(1,20) = 10.58, *p* = 0.004, $\eta_{p}^{2}=0.346$, *pw* = 0.871, indicating an enhancement in the aSL task performed under explicit than implicit conditions. The main effect of the length of exposure was also observed, *F*(1,20) = 5.16, *p* = 0.034, $\eta_{p}^{2}=0.205$, *pw* = 0.580, indicating, as in the case of children participants, a larger N100 amplitude in the second half than in the first half of the aSL tasks (Figure 4). In addition, the twofold aSL task × type of “word” interaction reached a marginally statistically significant level, *F*(1,20) = 4.31, *p* = 0.051, $\eta_{p}^{2}=0.117$, *pw* = 0.506. In this interaction, the effect of task was found for the high-TP “words,” showing a tendency for larger N100 amplitudes in the aSL task performed under explicit than implicit conditions (*p* = 0.001). In addition, the effect of type of “word” in the explicit condition showed a tendency for larger N100 amplitude for the high- than for the low-TP “words” (*p* = 0.039). Figure 2 depicts that effect.

#### N400

##### Children

Maximal effects were observed at the central ROI in this ERP component. The ANOVA showed the main effect of the aSL task, *F*(1,19) = 8.23, *p* = 0.010, $\eta_{p}^{2}=0.302$, *pw* = 0.777, indicating an enhancement in the aSL task performed under explicit than implicit conditions (Figure 3). In addition, the threefold type of “word” × aSL task × length of exposure was also significant, *F*(1,19) = 4.65, *p* = 0.044, $\eta_{p}^{2}=0.197$, *pw* = 0.535 (see Figure 4).

Pairwise comparisons showed that the effect of the aSL task resulted in a higher amplitude of the N400 component under explicit than implicit conditions, observed for low-TP “words” in the first half of the task (*p* = 0.030), while, in the second half of the task, that effect was observed for high-TP “words” (*p* = 0.027). Moreover, a significant effect of type of “word” was found in interaction with the aSL task and length of exposure, showing a larger amplitude for low-TP “words” relative to the high-TP “words” in the first half of the explicit aSL task (*p* = 0.041). Finally, the effect of length of exposure reached significance for low-TP “words” under explicit instructions, resulting in a larger N400 amplitude in the first half than in the second half (*p* = 0.022).

##### Adults

The analyses revealed a significant main effect of type of “word” at central ROI, *F*(1,20) = 6.88, *p* = 0.016, $\eta_{p}^{2}=0.256$, showing a larger N400 for the high-TP than for the low-TP “words” regardless of the aSL task (Figure 3). Moreover, the main effect of the length of exposure was also observed, *F*(1,20) = 8.15, *p* = 0.010, $\eta_{p}^{2}=0.289$, indicating an enhancement in the second than in the first half of the aSL tasks (Figure 3). No other main or interaction effect reached statistical significance.

## Discussion

The present study aimed to examine age-related differences in the neural correlates of aSL with linguistic materials during the familiarization phase of a triplet-embedded task. Five-year-old children and young adults were exposed to speech streams containing high- and low-predictable three-syllable nonsense words in which the statistical regularities had to be extracted through passive exposure (implicit condition) or after the nonsense words had been explicitly taught (explicit condition). The use of “words” with different levels of predictability aimed to increase the variance along which the aSL ability was measured and to mimic what occurs in “real” environments closely. The presentation of “words” under implicit and explicit conditions aimed to further examine if children take advantage of the previous knowledge to enhance SL, as previously observed with adult participants. As a whole, with this design, we aimed to contribute to a deepened understanding of the neurodevelopmental changes that the processes underlying aSL might undergo across development, and, ultimately, to test current views claiming for an invariant model of aSL with auditory linguistic materials.

Our findings support the view that aSL with linguistic materials changes through development. Behavioral data from adult participants showed 2-AFC performance exceeded the chance level in the aSL task performed under implicit and explicit conditions and for both types of “words,” except for the high-TP “words” in the implicit condition. However, in the group of children, the 2-AFC performance did not differ from chance in any condition. This disparity between children and adults’ results is consistent with recent works showing that aSL with non-linguistic materials, as well as with visual stimuli, improves with age (Arciuli and Simpson, 2011; Bertels et al., 2015; Lukács and Kemény, 2015; Raviv and Arnon, 2018; Shufaniya and Arnon, 2018; Emberson et al., 2019). Nonetheless, it is important to note that the differences in the 2-AFC performance across groups were made at the expense of the absence of reliable signs of learning in the group of children, hence recommending a more nuanced interpretation of the age-related differences in behavioral SL outcomes. Although the lack of behavioral signs of SL for children, even when explicit instructions were provided, might stem from the complexity of the speech streams used – which entailed a larger and more diverse number of triplets than in previous works – it is worth noting that these findings are in accordance with Raviv and Arnon’s (2018) and Shufaniya and Arnon’s (2018) studies, which did not find behavioral signs of SL for children below 6 years age [see also van Witteloostuijn et al. (2019) and Lukács et al. (2021) for similar results with 3-AFC tasks, and Soares et al. (2021d) for similar findings with the artificial learning paradigm]. Thus, more than a failure to track the statistical structure embedded in the input, what these behavioral results seem to indicate is that the 2-AFC task is not appropriate for assessing SL, particularly in children of this age, once they seem to lack the cognitive abilities needed to perform the 2-AFC task appropriately (e.g., see van Witteloostuijn et al. (2019), Arnon (2020), Lukics and Lukács (2021), Lukács et al. (2021)). Note that, to adequately discriminate a “word” from a foil in the 2-AFC task, participants need to use memory and metacognitive abilities that are not fully developed in children of this age (Gathercole et al., 2004). These factors might mask SL and may also justify why children seem not to take advantage of the previous knowledge of the to-be-learned regularities to boost 2-AFC performance, as observed with adult participants.

Another finding that deserves mention is the fact that, in the 2-AFC task performed under implicit conditions, the adults responded at chance in the case of high-TP “words,” conversely to what was observed for the low-TP “words.” This unexpected result, also observed recently by Soares et al. (2021c), can be accounted for if we attend to an inevitable consequence of the manipulation of words’ TPs in our stimuli, as well as in all the studies using triplets with different levels of predictability (see Bogaerts et al. (2016), Siegelman et al. (2018b), Johnson et al. (2020), Soares et al. (2020, 2021a,2021b), Gutiérrez-Domínguez et al. (2021), Lages et al. (2021)). Indeed, because high-TP “words” are made of unique syllables that occurred only in specific “words,” in specific syllable positions, conversely to low-TP “words,” whose syllables appeared in different “words” in different syllable positions (see section “Stimuli”), this might made the learning of the low-TP “words” to involve not only the encoding of a smaller number of syllables than high-TP “words” (12 vs. four, respectively), but, importantly, syllables that occurred three times more frequently in the stream than the syllables of the high-TP “words.” Thus, even though high- and low-TP “words” appeared exactly the same number of the times in the speech streams to account for “word” frequency effects in speech processing (see Soares et al. (2015, 2019) for a discussion), the fact that low-TP “words” entailed syllables that occurred more often, might have led participants, when asked to decide which of two stimuli “sounded more familiar” based on the stream presented before in the 2-AFC post-learning task, to choose the “words” that contained syllables that had occurred more frequently in the stream and that certainly generated higher levels of familiarity (see Soares et al. (2017) for a discussion of familiarity effects in word recognition).

Nevertheless, the results obtained from the ERP data provided evidence that both children and adults were able to extract the regularities embedded in the input as exposure to the speech streams unfolded, as indexed by modulations in the N100 and N400 components, taken as the neural signatures of SL (e.g., Sanders et al., 2002, 2009; Cunillera et al., 2006, 2009; De Diego Balaguer et al., 2007; Abla et al., 2008; Batterink and Paller, 2017; Soares et al., 2020). Specifically, larger N100 amplitudes were found in the second than in the first halves of the aSL tasks in both groups of the participants, as expected. Previous research has considered the N100 a “marker” of online segmentation in the brain (Sanders et al., 2002; Sanders and Neville, 2003; Abla et al., 2008), but the literature still presents divergent findings regarding how N100’s amplitude is modulated by specific factors (e.g., De Diego Balaguer et al., 2007; Cunillera et al., 2009). Our findings are in line with previous research showing enhancements in the N100 in the last part of the familiarization phase (Abla et al., 2008; Soares et al., 2020) and suggest that this ERP component indexes transient effects that change as learning/exposure to the stream unfolds. More importantly, they also suggest that an early brain mechanism of aSL is already present in 5-year-old children for the decoding of linguistic input. This evidence agrees with other works claiming that SL is an early-maturing skill supporting language acquisition (Saffran et al., 1996, 1997; see Romberg and Saffran (2010) for a review), even though a detailed analysis of the neural responses observed in adults vs. children participants suggests age-related differences in the processes recruited to extract the statistical regularities embedded in auditory streams implemented with linguistic materials.

Indeed, in adults, we found evidence of a larger N100 when the subjects were provided with prior knowledge of the “words” of the artificial language, a result not found with children. Given the early and sensory nature of this component, this might indicate that explicit learning mechanisms are already at play at this early stage of processing in adult participants, boosting the extraction of speech regularities, particularly for those sequences presenting high TPs. Although our study cannot determine the factors that may underlie this result, it is possible that children’s developing brains cannot recruit, at least as efficiently as adults’, the structures/circuits associated with explicit (declarative) knowledge (e.g., medial-temporal areas, including hippocampus) and that are known to improve with age (Turk-Browne et al., 2009; Karuza et al., 2013; Batterink et al., 2019). Nevertheless, an aSL task effect was found in children’s group in the N400 component, indicating larger amplitude for “words” presented under explicit than implicit conditions (as in the case of adults in the N100 component), hence suggesting that, in a later stage of processing, it is still possible to observe the effect that the prior knowledge played in enhancing online “word” segmentation in children’s developing brains. Interestingly, the threefold effect observed in the children’s group in this ERP component additionally showed that the effect of aSL task was observed for the low-TP “words” in the first part of the aSL task, and for the high-TP “words” in the last part of the task, which was not observed for adult participants. In adults, besides the effect of length of exposure (i.e., larger amplitudes in the second half vs. the first half of the aSL tasks) already observed in the N100 component, only the main effect of “word” type reached statistical significance in the N400 component. This effect showed that high-TP “words” elicited larger amplitudes than low-TP “words” regardless of the aSL task, hence supporting the view that this component can be taken in artificial learning paradigms as an index of the emergence of a pre-lexical trace of “words” in the brain (e.g., Sanders et al., 2002, 2009; Cunillera et al., 2006, 2009; De Diego Balaguer et al., 2007; Soares et al., 2020). The fact that high-TP “words” elicited larger N400 amplitudes than low-TP “words” as expected indicates not only that these “words” are more easily extracted from the input as observed in previous behavioral and EEG studies (e.g., Siegelman et al., 2018a; Soares et al., 2020), but also that the adult brain is able to decode the structure of continuous streams of syllables, distinguishing high-probable from less-probable sequences, even when “extra” (metalinguistic) information about the to-be-learned regularities was not provided.

In children, the recruitment of controlled and effortful processes for the processing of low-TP “words” during the first minutes of exposure can be accounted for if we assume that low-TP “words” are made up of syllables that are also found in other syllable sequences, hence producing less robust/stable perceptual representations (see Smalle et al. (2016) for interference effects produced by item overlap in a Hebbian repetition learning task). In the same vein, it is also possible to consider that the facilitation effect of explicit instructions observed in the first part of the aSL task for the low-TP “words” has allowed high-TP “words” to be automatically extracted (note that extracting one kind of “words” allows to automatically extract the other kind by bootstrapping). This would justify the pattern observed in the second part of the task for the high-TP “words.” Alternatively, it can also be argued that, unlike adults, children might have extracted the statistical regularities embedded in the input by using a simpler strategy, i.e., computing syllable frequency (i.e., the number of times a given syllable appeared in the speech stream) instead of the probability of one syllable to be followed by another syllable in the stream (i.e., TPs). This interpretation is supported by recent findings, suggesting that children learn better in unbalanced than balanced distributions (i.e., in Zipf distributions), as it occurs in natural languages (Lavi-Rotbain and Arnon, 2019, 2020, 2021). Due to cognitive limitations, the children’s immature brain might simply rely on the use of a more “economic” strategy, which may even have facilitated the learning of lower frequency elements later on (Bortfeld et al., 2005; Palmer et al., 2019; Lavi-Rotbain and Arnon, 2021; Soares et al., 2021a). Future research should contrast these two accounts by comparing the processing of homogenous speech streams (containing either low-TP or high-TP “words”) to heterogenous (mixed) streams, manipulating the frequency of occurrence of each token. They should also further test if extending the time of exposure would make children and adults show the same pattern of neural responses and behavioral results.

## Conclusion

The present study is, to the best of our knowledge, the first reporting ERP evidence of age-related differences in the mechanisms used by children and adults to extract word-like units from continuous speech streams. It highlights the usefulness of the ERP methodology to cope with the limitations of the offline post-learning tasks, particularly the 2-AFC task, and to compare groups of participants from different developmental stages. It also sheds light on how the mechanisms underlying aSL with linguistic materials might change across development as a function of “words” predictability and the conditions under which “words” are presented to the participants. Indeed, although 2-AFC data failed to show evidence of SL in children, even when explicit instructions were provided, the modulations observed in the N100 and N400 suggest that participants from both groups were sensitive to the regularities embedded in the speech streams. Nevertheless, the differences observed across groups in these components suggest that children and adults rely on different mechanisms to extract word-like units from speech streams, hence supporting the view that aSL with linguistic materials changes through development as has been observed in the auditory domain with non-linguistic materials, as well as in the visual domain.

## Data Availability Statement

The datasets presented in this study can be found in online repositories. The names of the repository/repositories and accession number(s) can be found in the article/Supplementary Material.

## Ethics Statement

The studies involving human participants were reviewed and approved by the University of Minho, SECSH 028/2018. Written informed consent to participate in this study was provided by the participants’ legal guardian/next of kin.

## Author Contributions

AS and LJ conceptualized the manuscript. F-JG-D, AL, and HO implemented the experiment and collected the data. F-JG-D analyzed the data. F-JG-D, MV, HO, and AS interpreted the data. AS and MV wrote the first draft of the manuscript. AS, LJ, F-JG-D, and MV critically revised the manuscript. HO and AL prepared the revised versions. All authors reviewed and approved the final version submitted.

## Conflict of Interest

The authors declare that the research was conducted in the absence of any commercial or financial relationships that could be construed as a potential conflict of interest.

## Publisher’s Note

All claims expressed in this article are solely those of the authors and do not necessarily represent those of their affiliated organizations, or those of the publisher, the editors and the reviewers. Any product that may be evaluated in this article, or claim that may be made by its manufacturer, is not guaranteed or endorsed by the publisher.
